# A Safety-First Mindset:  Role of Patient Safety Culture in Enhancing Healthcare Workers' Emotional Intelligence

**DOI:** 10.12688/f1000research.182767.1

**Published:** 2026-06-08

**Authors:** Shyamkumar Sriram, Nithya Priya, Bhoomadevi A, Malarvizhi Jayakumar

**Affiliations:** 1 Department of Rehabilitation and Health Services, University of North Texas, Denton, Texas, USA; 2 Faculty of Management Sciences, Sri Ramachandra Institute of Higher Education and Research(DU), Chennai, Tamil Nadu, 600019, India; 3 Public Health & Hospital Administration, Amity University, Noida, Uttar Pradesh, 201303, India; 4 College of nursing, Grand Valley State University Kirkhof College of Nursing, Allendale Charter Township, Michigan, USA

**Keywords:** Patient safety culture; Emotional Intelligence; Quality care, Good health and well-being, Incident reporting, handing over, medication error

## Abstract

**Background:**

Patient safety culture is vital in healthcare for providing a quality care. Emotional intelligence plays a very important role in managing the emotions of the healthcare professionals. This study focuses on impact of emotional intelligence on patient safety culture and importance of balancing the emotions of healthcare professionals.

**Objective:**

To analyze the impact of patient safety culture factors on emotional intelligence factors **.**

**Methods:**

The study focused on implementation of patient safety cultures and its impact on emotional intelligence. Methodology adopted in the study was descriptive study and stratified random sampling is used for data collection. The study analyzed the impact of patient safety on emotional intelligence factors. Statistics tools like frequency, correlation, regression was used to test the hypothesis

**Results:**

It was observed from correlation and regression that the factors like self- awareness, relationship management has a positive association on patient safety culture. It was found that integrating data through influencing factors significantly <0.01) improves the quality of care through patient safety.

**Conclusion:**

The study highlights the factors of patient safety culture like report errors, near misses, and safety concerns, facilitating a culture of transparency and improvement and its impact on self-awareness, relationship management. The study recommends the continuous training for healthcare professionals on managing the emotions.

## 1. Introduction

The Global Patient Safety Report states that there is a need for global collaboration and investment in patient safety to reduce the incidence rate and improve the quality of patient care delivery. ^1^ The primary objective is to prevent and mitigate the medical errors, incident’s that patients may encounter in the delivery of healthcare services. Improving event feedback mechanisms and communication of event-related improvements. ^2^

Across the globe, there is inadequate systems create an environment that increases the likelihood of errors or the failure to prevent them, resulting in adverse events. ^3^ Healthcare has been identified as a "high-hazard industry" due to its inherent risks of morbidity and mortality. ^4^ Evidence based guidelines to be followed effectively for avoiding the medical errors and to reduce the discharge delay process. ^5^ Improving the EI of employees will have positive job performance and reduces the burnout. ^6^ Emotional intelligence is crucial for improving clinical decision making ^7^

A majority of the available data originates from developed countries, prompting the World Health Organization. It encompasses the values, attitudes, perceptions, competencies, and behavioral patterns of an individual and group that influence the proficiency in health management. ^8^ Studies suggest that approximately 10% of patients may experience harm while receiving hospital care, and alarmingly, half of these incidents are considered preventable. ^9^ The World Health Organization (WHO) introduced a comprehensive approach to patient safety, conceptualized as a cycle.

Healthcare professional surveys have identified that feedback on patient safety culture is positive and has improved the functioning of hospital departments. ^10^ Overall, teamwork plays an important role and has a significant impact on team training, the productivity of healthcare providers, and communication between employees. ^11^ Most of the unit and hospital dimensions were correlated with the Safety Grade outcome measure in the tool. ^12^ Event reporting, communication, patient safety leadership and management, staffing were identified as major patient safety culture predictors. ^13^

**Research Questions:**

1. What is the impact of emotional intelligence on patient safety culture?
2. What is relationship between occupation and emotional intelligence?

## 2. Materials and methods

### 2.1 Research design

This quantitative approach was exhibited in the study to evaluate the perceptions of various demographic groups regarding patient safety culture and emotional intelligence. It also explored the relationship between patient safety culture and emotional intelligence, as well as the impact of patient safety culture on emotional intelligence.

### 2.2 Study sites/settings and populations

Three private multi-specialty hospitals were involved in the study. Probability sampling was used to collect the data. Probability sampling included stratified random sampling, that divides the study population into different categories and selecting a random sample from each category, resulting in more accurate selection of samples. Healthcare professionals (HCPs) were selected from diverse backgrounds, representing various cadres and holding different designations and positions within the hospital. The HCPs were categorized into three distinct groups or strata: hospital administrators, nursing staff, and paramedical staff (including pharmacists, lab technicians, dialysis staff, and operation theatre technicians). The sample frame consisted of 640 respondents (nurses, technical staff, and hospital administrators) from the three private multi-specialty hospitals. A well-designed questionnaire was administered among 100 respondents to test the reliability using Cronbach’s alpha for all dimensions of the study. Based on the findings of the pilot study, the questionnaires were reformulated for better results. Regression findings highlighted significant association between patient safety outcomes and composites. ^14^

### 2.3 Participants

Six hundred forty respondents participated in the study to evaluate the relationship between patient safety culture and emotional intelligence. Table 1 shows that the respondents consisted of 35.6 percent of healthcare professionals in the 25–34 age group, 42.7 percent in the 35–44 age group, 11.6 percent in the 45–54 age group, and 10.2 percent above 55 years of age. Gender-wise, 51.9 percent were female healthcare professionals and 48.1 percent were male. Of the healthcare professionals, 56.7 percent were married and 43.3 percent were unmarried. Among healthcare professionals, 54.4 percent were nurses, 21.1 percent were technical staff, and 24.5 percent were hospital administrators. The monthly income was below 30,000 for 40.8 percent and above 70,000 for 6.4 percent. It was also observed that 29.1 percent had up to 5 years of experience, 34.7 percent had between 6–10 years, 19.1 percent had 11–15 years, and 17.2 percent had more than 15 years of experience.

**Table 1.** Demographic profile.

S. No	Demographic Variable		Frequency	Percentage %
1.	**Age Group in years**	25–35	228	35.6
		35–45	273	42.7
		45–55	74	11.6
		Above 55	65	10.2
2.	**Gender**	Male	308	48.1
		Female	332	51.9
3.	**Marital Status**	Married	363	56.7
		Unmarried	277	43.3
4.	**Monthly Income**	Below 30000	261	40.8
		30000–50000	238	37.2
		50000–70000	100	15.6
		Above 70000	41	6.4
5.	**Occupation**	Nurses	348	54.4
		Technician Staff	135	21.1
		Hospital Administration	157	24.5
6.	**Experience**	Upto 5	186	29.1
		6–10	222	34.7
		11–15	122	19.1
		Above 15	110	17.2

Source: Primary data.

### 2.4 Study tools/instruments

The study participants completed a self-report questionnaire, which included patient safety culture questions developed by the Agency for Healthcare Research and Quality (AHRQ). The Hospital Survey on Patient Safety Culture (HSOPSC) was developed by the US Agency for Healthcare Research and Quality (AHRQ). The questionnaire for emotional intelligence factors was developed from the study "Relationship between Emotional Intelligence and Patient Safety Culture" ^15^ and Daniel Goleman's Emotional Intelligence Framework. Based on the study findings, it was determined that the Indian version (used in this study) demonstrated good validity and acceptable reliability, with Cronbach’s alpha values ranging from 0.51 to 0.73. Therefore, it was deemed an appropriate instrument for evaluating patient safety culture and emotional intelligence in Indian public hospitals.

### 2.5 Informed consent

Informed consent was obtained from the healthcare professionals. Since the respondence group was healthcare employees verbal informed consent was obtained before collecting the data.

### 2.6 Data collection

Both primary and secondary data were collected in the present study. Primary data was collected using a well-defined questionnaire with the sample size of 640.The data collection period was from January 2022 to July 2022, spanning six months in each hospital. Data were collected from nurses in General Medicine, Cardiology, Operation Theatre, Gynaecology, and Nephrology departments, as well as from hospital administrators and technical staff (including pharmacists, lab technicians, dialysis staff, and operation theatre technicians). This study was approved by IEC board of SRIHER (DU) and the IEC number is IEC-NI/21/FEB/77/06.

### 2.7 Data analysis

The statistical techniques used in the study were aligned with the research objectives, which aimed to determine the relationships between variables. Data were analyzed using the Statistical Package for Social Sciences (SPSS Version 16). The study used correlations to examine inter-correlations among various patient safety dimensions, emotional intelligence, and organizational factors. Assuming a type I error of 5% and 60% of good perception responses, the minimum sample size calculated was 640 respondents across the three multi-specialty hospitals. The t-test was used to test hypotheses regarding differences in gender, marital status, age, income group, and experience with patient safety culture dimensions. Professor R.A. Fisher is credited with coining the term 'variance' and pioneering the theory of Analysis of Variance (ANOVA), which elucidated its practical utility. Multivariate analysis refers to a set of statistical techniques used to analyze relationships between multiple variables simultaneously. All statistical tests were considered significant at a p-value of less than 0.05. The internal validity of the instruments was assessed using Cronbach’s alpha.

## 3. Results

A detailed analysis of the collected data was conducted in accordance with the objectives stated earlier. Hypotheses were also tested based on the findings of the study, and interpretations and conclusions were drawn. In this chapter, various statistical techniques were used for data analysis, including descriptive analysis and inferential statistics. Hypotheses were framed based on dependent and independent variables.

Table 1 shows the demographic profile of the respondence that includes age, gender, marital status, income, occupation and experience. And it was observed that majority of the respondence were female and nurses with experience of 6-10 years.

Null Hypothesis (H _0_):
**There is no difference among the occupations of the respondents regarding emotional intelligence factors.**

As shown in Table 2, there is a significant difference between the occupations of respondents with regard to self-awareness, self-management, relationship management, and overall emotional intelligence. Since the p-value is less than 0.05, the null hypothesis is rejected at the 5% level with regard to experience and social consciousness. Hence, there is a significant difference between occupations with regard to social consciousness.

**Table 2.** Occupation of the respondents towards emotional intelligence factors.

S. No	EmotionalIntelligence Factors	Occupation			F value	P value
		Nurses	Technical staff	Hospital Administrators		
		Mean (SD)	Mean (SD)	Mean (SD)		
1.	Self-Awareness	15.41 (3.98)	16.53 ^b^ (3.59)	16.3 ^b^ (3.85)	5.837	0.003**
2.	Self-Management	13.23 ^a^ (3.19)	14.13 ^b^ (3.11)	13.97 ^b^ (3.11)	5.386	0.005**
3.	Social Consciousness	17.31 ^a^ (3.33)	18.14 ^b^ (3.39)	17.59 ^b^ (3.30)	3.051	0.048*
4.	Relationship Management	16.60 ^a^ (4.02)	18.36 ^b^ (3.73)	17.68 ^b^ (3.95)	11.016	0.000**

Source: Primary data.

Incidence reporting was influenced by feedback and communication about error, staff position, teamwork across units, non-punitive response to error, supervisor/managers expectations and actions promoting patient safety. ^16^ The results show a positive correlation among patient safety culture factors. This finding is consistent with research by Dr. Beatrice J. Kalisch et al. (2011), who stated that when patients stay longer in wards, teamwork across and within units improves. Higher levels of patient care are provided with adequate staffing. Inadequate communication regarding medication details—such as name, dosage, route of administration, and timing—between physicians, pharmacists, nurses, and patients can result in medication error. ^17^ Adverse events can be identified using Electronic Medical Records to overcome various issues. ^9^ Awareness of the nurses regarding patient safety should be enhanced. ^18^

Table 3 shows that the correlation coefficient between patient safety culture and emotional intelligence factors is positive and significant at the 1% level. It was observed that the correlation between teamwork within units and relationship management is 0.63, indicating a 63% positive correlation. This is supported by a study conducted by ^19^ which found that healthcare teams encounter prevalent challenges including accountability, conflict management, decision-making, reflection on progress, and coaching. Additionally, the correlation between a non-punitive response to error and self-awareness is 0.43, indicating a 43% positive correlation. ^20^ observed a significant improvement in the dimension of "hospital management support for patient safety," with all main effects found to be statistically significant. It is revealed that customized approach for each professional group can implement patient safety strategy. ^21^ Improvement in patient safety culture will reduce adverse event. ^22^ Nurses are important communicators; specially about hospital safety and quality. ^23^ The feedback on patient safety culture stimulated actions to improve patient safety culture. ^24^

**Table 3.** Correlation analysis.

Factors of Patient Safety Culture	Self-Awareness	Self-Management	Social Consciousness	Relationship Management
**Teamwork within units**	0.510 **	0.552 **	0.586 **	0.638 **
**Supervisor/Manager Expectations**	0.498 **	0.516 **	0.496 **	0.607 **
**Organizational Learning**	0.499 **	0.551 **	0.573 **	0.587 **
**Management support**	0.500 **	0.492 **	0.536 **	0.566 **
**Perceptions**	0.489 **	0.520 **	0.541 **	0.602 **
**Feedback and Communication about Error**	0.485 **	0.552 **	0.561 **	0.613 **
**Communication Openness**	0.546 **	0.582 **	0.558 **	0.568 **
**Frequency of Events Reported**	0.546 **	0.516 **	0.551 **	0.540 **
**Teamwork Across Units**	0.446 **	0.570 **	0.478 **	0.575 **
**Staffing**	0.550 **	0.537 **	0.572 **	0.627 **
**Handoffs and Transitions**	0.528 **	0.507 **	0.500 **	0.537 **
**No punitive Response to Errors**	0.438 **	0.570 **	0.523 **	0.619 **

Source: Primary data.

** Denotes significant at 1% level.

Table 4 shows that the multiple correlation coefficient is 0.894, measuring the degree of relationship between the actual values and the predicted values of overall emotional intelligence. The coefficient value of 0.894 indicates that the relationship between overall emotional intelligence and the twelve independent variables of patient safety culture is quite strong and positive. The coefficient of determination, R-square, measures the goodness-of-fit of the estimated Sample Regression Plane (SRP). The value of R-square is 0.799, which means that about 79.9% of the variation in overall emotional intelligence is explained by the estimated SRP that uses patient safety culture variables.

**Table 4.** Variables of multiple regression analysis.

**Multiple R value**	0.894
**R Square value**	0.799
**F value**	201.168
**P value**	<0.001 **

Variables	Unstandardized co-efficient	SE of B	Standardized co-efficient	t value	P value
Constant	7.327	1.466		4.997	<0.000 **
Teamwork within Units (X1)	0.718	0.116	0.161	6.182	<0.000 **
Supervisor/Manager Expectations (X2)	0.269	0.091	0.076	2.948	0.003 **
Organizational Learning (X3)	0.450	0.124	0.096	3.631	<0.000 **
Management Support (X4)	0.392	0.155	0.065	2.528	0.012 **
Perceptions of patient safety culture (X5)	0.252	0.116	0.057	2.168	0.031 **
Feedback and communication about errors (X6)	0.363	0.099	0.099	3.664	<0.000 **
Communication Openness (X7)	0.474	0.120	0.108	3.964	<0.000 **
Frequency of Events Reported (X8)	0.679	0.120	0.148	5.674	<0.000 **
Teamwork Across Units (X9)	0.269	0.093	0.071	2.888	0.004 **
Staffing (X10)	0.468	0.095	0.135	4.934	<0.000 **
Handoffs and Transitions (X11)	0.191	0.111	0.044	1.718	0.086 **
No punitive Response to Errors (X12)	0.391	0.099	0.110	3.957	<0.000 **

Source: Primary data.

** Denotes significant at 1% level.

The multiple regression equation is

$$Y=7.327+0.71{\text{8X}}_{1}+0.26{\text{9X}}_{2}+0.45\text{0X3}+0.39\text{2X4}+0.25\text{2X5}+0.36\text{3X6}+0.47\text{4X7}+0.67\text{9X8}+0.26\text{9X9}+0.46\text{8X}10+0.19\text{1X}11+0.39\text{1X}12$$

The coefficient of teamwork within units (X _**1**_ **)** is 0.718 represents the partial effect of teamwork within units on overall emotional intelligence, holding the other variables as constant. the estimated positive sign implies that such effect is positive that overall emotional intelligence would increase by 0.718 for every unit increase in teamwork within units and this coefficient value is significant at 1% level and thus, all the patient safety variables are significant at 1% level. Based on standardized coefficient, teamwork within units (0.161) is the most important factors to extract overall emotional intelligence, followed by frequency of events reported (0.148), staffing (0.135), no punitive response (0.110), feedback and communication about errors (0.108), communication openness (0.099), organizational learning (0.096), supervisor/manager expectations (0.76), great teamwork across units (0.071), management support (0.065), handoffs and transitions (0.044). Teamwork plays a vital role in managing the emotions of patients.

## 4. Discussion

This research highlights the significant impact of patient safety culture on emotional intelligence factors. The main finding of this study is that the emotions of healthcare professionals influence patient safety culture practices. Statistical analysis of various demographic factors showed that there is no significant difference between gender and staffing. However, the perception of patient safety culture among age groups with up to 5 years and 6–10 years of experience differs from those with 11–15 years and more than 15 years of experience. These results correspond with the work ^25^ who assessed the perception of patient safety culture in Saudi Arabia. Studies reveal that improper staffing leads to medical errors and increased patient safety incidents ^26^ found that adequate physician staffing was linked to improved patient outcomes, such as reduced complications, fewer adverse events, and better overall quality of care, while insufficient physician staffing was associated with increased mortality rates, higher complication rates, and poorer patient outcomes.

It was found that female staff communicate more effectively than male staff, which is supported by, ^27^ who studied gender differences in medical encounters. It was also found that overall emotional intelligence would increase by 0.718 for every unit increase in teamwork within units, and this coefficient value is significant at the 1% level.

The correlation coefficient between handoffs and transitions and respondents’ emotional intelligence would increase by 0.191 for every unit increase in handoffs and transitions, though this coefficient value is not significant at the 1% level. This finding corresponds with other research carried out by ^3^ (2015), which found that physicians with high emotional intelligence experience enhanced job satisfaction, reduced burnout, improved patient-physician relationships, increased patient satisfaction, and demonstrate greater effectiveness as leaders and communicators. According to the findings of, ^5^ safety culture significantly influenced important healthcare outcomes, including medication errors, back injuries, and patient satisfaction.

It was identified that the correlation coefficient between self-awareness and policies is 13.17%, indicating a positive relationship that is significant at the 1% level and suggests room for improvement. This is similar to the findings of ^25^ who found a correlation between emotional intelligence (EI) and team performance. Teams with high emotional intelligence can effectively understand and interpret each other's cues, leading to improved communication and a reduction in miscommunication. Hence, there is a significant difference between the experiences of respondents regarding self-management. The correlation coefficient between self-management and social consciousness is 24.6%, indicating a positive relationship that is significant at the 1% level.

This is similar to the study conducted by, ^26^ which found that the capacity to effectively handle and comprehend emotions enhances patient safety and is recognized as a critical skill for healthcare professionals. This finding also corresponds with research by, ^27^ which showed that physicians with high emotional intelligence experience enhanced job satisfaction, reduced burnout, improved patient-physician relationships, increased patient satisfaction, and demonstrate greater effectiveness as leaders and communicators.

## 5. Conclusions

This study emphasizes the impact of patient safety culture on emotional intelligence and its role in patient care delivery. The study also highlights existing reviews on patient safety culture practices, emotional intelligence, and organizational factors. The WHO emphasizes the need for a just culture that focuses on learning from errors rather than blaming individuals. Establishing a non-punitive approach encourages healthcare professionals to report errors, near misses, and safety concerns, facilitating a culture of transparency and improvement. It is reported that around 1 in 10 hospitalized patients experience harm, with at least 50% of these incidents being preventable. Around two-thirds of all adverse events resulting from unsafe care—and the years lost to disability and death—occur in low- and middle-income countries (LMICs).

The study concludes that dimensions such as Communication Openness, Supervisor/Manager Expectations, Handoffs and Transitions, Organizational Learning, Self-Awareness, and Relationship Management need greater focus compared to other dimensions of patient safety culture. It was also found that psychological interventions can enhance the self-awareness of employees. Such interventions are effective in reducing anxiety and helping healthcare professionals understand the emotional state of patients. In conclusion, this study provides valuable insights into the importance of patient safety culture and its impact on emotional intelligence factors.

## 6. Implications

The findings of this research highlight the impact of patient safety culture on emotional intelligence. Implementing these managerial actions can enhance patient safety practices. Encouraging healthcare providers to report incidents will improve the identification of near misses and unsafe conditions. Effective incident reporting also improves transparency and accountability. Furthermore, incident reporting can mitigate the emotional and psychological impact on healthcare professionals involved in adverse events and promote their well-being.

Centralizing patient data helps reduce the risk of errors, such as duplicate tests or conflicting medications. Healthcare providers can review the complete medical history, including allergies, previous diagnoses, and treatments, which enables them to make informed decisions, avoid adverse events, and improve patient safety.

Emotional intelligence training emphasizes the development of empathy and compassion, enabling healthcare professionals to understand and connect with patients on an emotional level.

## Preregistered data analysis

The study data was not preregistered the research with data analysis plan at an independent registry

## Clinical trial

Not Applicable

## Informed consent

Informed consent was obtained from the healthcare professionals. Since the respondence group was healthcare employees verbal informed consent was obtained before collecting the data.

## Ethical approval statement and consent for participation

**Ethical consideration:** Ethical clearance was obtained from IEC of Sri Ramachandra Institute of Higher Education and Research for the conduct of the study with the following ethics clearance number IEC-NI/21/FEB/77/06.

Since the respondent were healthcare professionals. There is no confidential data collected, therefore consent forms are not collected.

## Authors’ contribution

Conceptualization, A.BD.; methodology, N. P. S.; software, S.K.S; validation, A.BD., N. P. S., and B.D.A.; formal analysis, investigation, resources, data curation, S.K.S., and N. P. S. Writing —original draft preparation, M. J and N. P. S.; writing—review and editing, S.K.S; visualization, M. J, A.B.D and S.K.S; supervision and project administration, M. J, N.P.S. and A.B.D. All authors have read and agreed to the published version of this manuscript.

## Data Availability

Since patient related information were collected in the study from the healthcare professionals, the access to data is restricted as per the norms of IEC board. Due to the sensitive nature of the questions asked in this study, survey respondents were assured raw data would remain confidential and would not be shared. But, upon request to the corresponding author nithya003priya@gmail.com the data set can be provided for genuine reasons.
