# Supplementary material for: A Safety-First Mindset:  Role of Patient Safety Culture in Enhancing Healthcare Workers' Emotional Intelligence
Source: F1000Res. 2026 Jun 8;15:883. [Version 1] doi: 10.12688/f1000research.182767.1 (PMC13324356; doi:10.12688/f1000research.182767.1)
Supplement: Peer review history

# Reviewer report: Reviewer response for version 1

**DOI:** 10.5256/f1000research.201735.r497318

**Published:** 2026-07-02

**Authors:** Amutha Anandakumar

**Affiliations:** 1 Department of Childhood and Youth Onset Diabetes, Madras Diabetes Research Foundation (ICMR Collaborating Centre of Excellence), Chennai, Tamil Nadu, India

Thank you to the Editorial Board for sending me the manuscript for review. The authors have taken an interesting topic of how emotions of health care professionals influence patient safety culture practices, and the manuscript has been written well. The format and organization of the manuscript, introduction, methods, statistical analysis, results, tables, and discussion were done meticulously. The following minor edits will provide clarity, and the manuscript may be **“Accepted”** upon their implementation.

 1. Please specify the location of the three private multi-specialty hospitals that were involved.

 2. The data collected by the authors were primary data. No need to mention that secondary data were collected.

 3. A line on future research perspectives can be added.

Is the work clearly and accurately presented and does it cite the current literature?

Yes

If applicable, is the statistical analysis and its interpretation appropriate?

Yes

Are all the source data underlying the results available to ensure full reproducibility?

Yes

Is the study design appropriate and is the work technically sound?

Yes

Are the conclusions drawn adequately supported by the results?

Yes

Are sufficient details of methods and analysis provided to allow replication by others?

Yes

Reviewer Expertise:

Health care

I confirm that I have read this submission and believe that I have an appropriate level of expertise to confirm that it is of an acceptable scientific standard.

# Reviewer report: Reviewer response for version 1

**DOI:** 10.5256/f1000research.201735.r494947

**Published:** 2026-07-01

**Authors:** Abeetha S

**Affiliations:** 1 ACS Medical College and Hospital, Chennai, Tamil Nadu, India

The Manuscript addresses an important topic by exploring the relationship between emotional intelligence and patient safety culture among Health care workers. The subject is relevant to improving healthcare quality and performance of the organization. I would suggest a minor revision: 

 1. Correct grammatical errors and improve sentence construction throughout the manuscript.

 2. Replace vague statements such as "integrating data through influencing factors" with clearer explanations.

Is the work clearly and accurately presented and does it cite the current literature?

Yes

If applicable, is the statistical analysis and its interpretation appropriate?

Yes

Are all the source data underlying the results available to ensure full reproducibility?

Yes

Is the study design appropriate and is the work technically sound?

Yes

Are the conclusions drawn adequately supported by the results?

Yes

Are sufficient details of methods and analysis provided to allow replication by others?

Yes

Reviewer Expertise:

Medical education, Non communicable diseases, Exercise and Yoga

I confirm that I have read this submission and believe that I have an appropriate level of expertise to confirm that it is of an acceptable scientific standard.

# Reviewer report: Reviewer response for version 1

**DOI:** 10.5256/f1000research.201735.r497317

**Published:** 2026-06-30

**Authors:** K. C. Raja Shree

**Affiliations:** 1 Saveetha School of Management, Saveetha Institute of Medical and Technical Sciences, Chennai, Tamil Nadu, India

The manuscript is well organized and addresses an important research topic. The objectives, methodology, and findings are presented clearly. However, the paper would benefit from the inclusion of more recent scholarly references (preferably from the last 3–5 years) to strengthen the literature review and support the discussion with current evidence. In addition, the authors are encouraged to include a separate section on practical recommendations, particularly highlighting the implications of the findings for the healthcare sector. This would enhance the practical relevance, applicability, and overall contribution of the study. The manuscript may be considered for indexing after these minor revisions are addressed.

Is the work clearly and accurately presented and does it cite the current literature?

Yes

If applicable, is the statistical analysis and its interpretation appropriate?

Yes

Are all the source data underlying the results available to ensure full reproducibility?

Yes

Is the study design appropriate and is the work technically sound?

Yes

Are the conclusions drawn adequately supported by the results?

Yes

Are sufficient details of methods and analysis provided to allow replication by others?

Yes

Reviewer Expertise:

Healthcare Management

I confirm that I have read this submission and believe that I have an appropriate level of expertise to confirm that it is of an acceptable scientific standard.
